# Recent Insights into the Molecular Mechanisms of Salt Tolerance in Melon (*Cucumis melo* L.)

**DOI:** 10.3390/plants14233598

**Published:** 2025-11-25

**Authors:** Yanping Jing, Jihai Yang, Dingfan Xu, Qiufeiyang Chen, Kexing Xin, Xunfeng Chen, Jun Tang, Jian Chen, Zhihu Ma

**Affiliations:** 1 School of Life Sciences, Jiangsu University, Zhenjiang 212013, China; 3234501016@stmail.ujs.edu.cn (J.Y.); 3234501005@stmail.ujs.edu.cn (Q.C.); xinkexing@ujs.edu.cn (K.X.); jianchen@ujs.edu.cn (J.C.); 2 Zhenjiang Institute of Agricultural Sciences in Hilly Area of Jiangsu Province, Jurong 212400, China; 20230060@jaas.ac.cn; 3 Key Laboratory of Zhenjiang, School of Environment and Safety Engineering, Jiangsu University, Zhenjiang 212013, China; xunfengchen@ujs.edu.cn; 4 Jiangsu Provincial Key Laboratory of Agrobiology, Institute of Germplasm Resources and Biotechnology, Jiangsu Academy of Agricultural Sciences, Nanjing 210014, China; 20220041@jaas.ac.cn

**Keywords:** *Cucumis melo* L., salt stress, ion homeostasis, antioxidant defense, transcriptional regulation

## Abstract

Salt stress represents one of the most critical abiotic constraints limiting global agricultural productivity by adversely affecting plant growth, metabolism, and yield. Soil salinization disrupts water uptake and nutrient homeostasis, leading to ionic toxicity, osmotic imbalance, and oxidative stress that collectively impair crop development. *Cucumis melo*, a major horticultural crop of significant economic value, exhibits high sensitivity to salinity. Recent advances have elucidated that melon adapts to salt stress through intricate physiological and molecular mechanisms involving osmotic adjustment, ion transport regulation, antioxidant defense, and transcriptional reprogramming. Several pivotal genes, such as *CmNHX1*, *CmHKT1;1*, *CmCML13*, *CmAPX27*, and *CmRAV1*, etc., have been identified to participate in multiple signaling pathways governing salt tolerance in melon. In this review, we comprehensively summarize the physiological effects of salt stress on melon growth, elucidating the key molecular mechanisms underlying salt tolerance, particularly those associated with ion homeostasis, antioxidant defense, and transcriptional regulation. The review further discusses current strategies and future perspectives for the genetic improvement of salt tolerance. Collectively, this review provides a theoretical framework and valuable reference for future research on the molecular basis of salt tolerance and breeding of salt-tolerant melon cultivars.

## 1. Introduction

*Cucumis melo* L. (melon), a member of the *Cucurbitaceae* family, is widely cultivated in warm climates worldwide, prized for its flavorful fruits, varied morphologies, and substantial economic contributions to horticulture. Recent increases in soil salinity and water scarcity in traditional melon-growing regions pose a significant threat to its production. Elevated soil salt levels trigger osmotic stress, ionic toxicity, and oxidative damage, severely constraining melon productivity in saline and semi-arid environments.

Soil salinization imposes complex constraints on plants by causing osmotic stress (reduced water uptake), ion toxicity (particularly high Na^+^ and Cl^−^), nutrient imbalances (e.g., K^+^/Na^+^ ratio disruption), and oxidative damage [1]. In melon, these stresses manifest as reductions in growth, root and shoot development, leaf area, photosynthetic capacity and fruit yield and quality [2]. Physiological and biochemical studies have revealed genotype-specific responses, for instance, in K^+^/Na^+^ ratio, amino acid accumulation (phenylalanine, proline, histidine), and stress-induced metabolite changes between salt-tolerant and salt-sensitive cultivars [3].

Despite the growing awareness of the impact of salt stress on melon, the crop remains relatively sensitive compared with its more tolerant *Cucurbitaceae* relatives, and substantial knowledge gaps remain regarding its molecular response mechanisms (e.g., ion transporters, transcriptional regulators, signaling pathways) and how these could be exploited for breeding [4]. Recent studies indicate that among *Cucurbitaceae* crops, pumpkin and melon display relatively better salinity tolerance compared to sensitive species like cucumber, though comprehensive mechanistic work remains limited [4].

Given this background, this review aims to (i) summarize the principal physiological effects of salt stress on melon growth and development; (ii) present the molecular mechanisms underlying melon salt tolerance, focusing on ion homeostasis, antioxidant defense, and transcriptional regulation; and (iii) discuss current breeding or biotechnological strategies for enhancing salt tolerance in melon germplasm and propose future perspectives for research. Collectively, this review provides an integrative framework for understanding the physiological and molecular basis of salt tolerance in melon and offers theoretical and practical guidance for the breeding of salt-tolerant cultivars.

## 2. Salt Stress Damage in Melon

### 2.1. Germination and Seedling Growth Inhibition

Salt stress significantly impairs melon (*Cucumis melo* L.) seed germination and seedling development, with effects varying by cultivar and salt concentration. High salinity (≥100 mM NaCl) has been reported to markedly suppress melon seed-germination parameters, including germination rate, potential, index and speed [5]. The extent of inhibition varies substantially among cultivars [5,6]. For example, several cultivars such as Xindongfangmi and Jinyuliuxing showed salt tolerance, with germination rates above 90% under 200 mM NaCl treatment; however, other cultivars such as Jintiancui and Huaxiami were sensitive to NaCl treatment during seed germination [4]. As salinity intensifies, germination becomes increasingly delayed or may even be prevented altogether, principally due to osmotic stress that restricts water uptake and interferes with the metabolic processes required for germination [7]. For instance, high salinity inhibits the activity of key reserve-mobilizing enzymes such as α-amylase, which are critical for breaking down stored starch and supplying energy during germination, thereby limiting seeds from achieving full germination potential in rice and barley [8,9].

Seedling development of *Cucumis melo* is also markedly compromised under salt stress, showing pronounced declines in radicle length, hypocotyl elongation and seedling biomass, and the severity of inhibition is largely dependent on salt concentration and cultivar differences [3,5]. The root system, being the most sensitive organ to saline conditions, undergoes significant growth suppression, as salt concentration increases, root length, root volume, biomass and overall root growth decline progressively [4,5,10,11]. Such reductions in root vigor directly impair water and nutrient uptake, thereby limiting translocation to above-ground organs and consequently reducing photosynthetic capacity and whole plant growth [4,5,11]. In addition, salt-induced changes in root morphology, such as reduced root branching and diminished lateral root growth, further restrict the plant’s ability to explore the soil for water and essential nutrients [12].

### 2.2. Fruit Quality and Yield Reduction

Salt stress significantly affects melon fruit quality and yield, with the relationship between salinity levels and these outcomes being complex and non-linear [2,13,14]. At mild salinity levels, when the soil salinity is usually below 4 dS/m, the fruit quality in melon often improves due to enhanced accumulation of soluble sugars, total soluble solids (TSS), and vitamin C [2,13,14]. These improvements result from osmotic adjustment, where plants accumulate compatible solutes to maintain turgor pressure and stabilize enzymatic activity, thereby increasing sweetness and marketability [2,13,14]. However, it is important to note that such quality improvements may come at the cost of reduced vegetative growth or decreased fruit yield, reflecting a common trade-off between enhanced TSS and overall productivity under mild salinity. When salinity exceeds critical thresholds (typically >100 mM NaCl), these benefits diminish. Prolonged exposure to high salinity disrupts nutrient transport and metabolic homeostasis, leading to reduced fruit growth, smaller longitudinal and transverse diameters, and a significant decline in individual fruit weight [3,14,15,16,17]. Furthermore, nutritional quality declines due to oxidative stress and impaired biosynthesis pathways, which reduce vitamin C and carotenoid contents [2]. Field trials indicate that severe salinity (e.g., 6.1 dS m^−1^) can decrease marketable yield by 12–39%, although genotypic variation suggests that some melon cultivars (such as the Galia cultivar) exhibit greater resilience [13].

### 2.3. Physiological Stress

#### 2.3.1. Osmotic Stress

Salt stress in plants induces severe osmotic challenges by elevating soil osmotic pressure, which lowers water potential and impairs water uptake, effectively creating a physiological drought [1]. When the osmotic potential of the soil solution becomes more negative than that of plant cells, water uptake is severely restricted, leading to cellular dehydration, loss of turgor, and, in severe cases, plant death [18]. This osmotic stress is particularly critical during seed germination and early seedling development, as high NaCl concentrations hinder water imbibition, radicle emergence, and cell expansion, ultimately reducing seed vigor and suppressing subsequent growth [1,18]. For instance, melon seed germination is completely inhibited at an osmotic potential of −0.5 MPa and significantly reduced (up to 90%) at −0.4 MPa due to limited water absorption [19]. During seedling development, osmotic stress reduces stomatal conductance, transpiration, and photosynthetic efficiency, thereby restricting biomass accumulation. Comparative studies have shown that under salt stress, leaf water potential (1.12–1.25 MPa) is markedly lower than under drought stress (1.3–2.67 MPa) in melon, underscoring the osmotic component of salinity-induced growth inhibition [3]. Similarly, investigations in four melon genotypes under 200 mM NaCl revealed reduced stomatal conductance, likely a protective response to conserve water, alongside decreased leaf water and osmotic potentials, highlighting osmotic-driven cell dysfunction [20].

To counteract osmotic stress, plants accumulate a range of compatible osmolytes, including proline, soluble sugars, glycine betaine, soluble proteins, and polyols, which help maintain cellular osmotic balance and protect macromolecular structures [21,22]. These metabolites contribute to lowering cellular osmotic potential, thereby sustaining water uptake, maintaining turgor, and stabilizing membranes under saline conditions. For example, exogenous proline application effectively alleviates NaCl-induced salt stress in the two melon varieties Yuhuang and Xuemei [23].

Beyond their role in osmotic adjustment, these osmolytes also protect against oxidative damage by scavenging reactive oxygen species (ROS), which accumulate due to disrupted ion homeostasis and reduced photosynthetic efficiency [3,19]. Salt-induced disruption of ion homeostasis and impairment of photosynthesis often result in excessive accumulation of ROS, which can damage lipids, proteins, and nucleic acids, ultimately leading to oxidative stress and cellular injury [1,18]. Osmolytes such as proline act as molecular chaperones and ROS scavengers, thereby preserving enzyme activity, protecting photosynthetic machinery, and enhancing overall cellular redox balance [21,22,23]. In this way, osmolyte accumulation represents a dual defense mechanism in melon, simultaneously ensuring water balance and mitigating oxidative damage under salinity stress.

#### 2.3.2. Ionic Toxicity and Nutrient Imbalance

Ionic toxicity in plants primarily arises from the excessive accumulation of Na^+^ and Cl^−^ ions, which disrupt cellular homeostasis and metabolic processes [1]. Under saline conditions, Na^+^ influx into melon roots competes with essential cations like K^+^, leading to ion imbalances that manifest as reduced growth, yield losses, and fruit quality degradation [1]. For instance, in salt-sensitive melon genotypes, high Na^+^ levels in leaves and roots cause membrane depolarization and enzyme inhibition, while Cl^−^ accumulation damages chloroplasts, reducing chlorophyll content and photosynthetic efficiency [24]. This toxicity is evident in studies where salt-tolerant melon landraces, such as Huangdanzi and Zajiaojiashigua cultivars, exhibit lower Na^+^ content in young leaves compared to sensitive ones like Akekekouqi and Paodangua cultivars, highlighting Na^+^ exclusion as a key tolerance mechanism [25]. Nutrient imbalances further compound these effects; Na^+^ antagonism inhibits K^+^ uptake, lowering the K^+^/Na^+^ ratio, which is critical for osmotic regulation and enzyme activation in melons [3]. Salt-tolerant melon cultivars maintain higher K^+^/Na^+^ ratios under high salt stress, with distinctive traits like elevated proline and histidine levels aiding in mitigating ionic stress [3]. For instance, in grafted melons, overexpression of genes such as *CmDUF239-1* enhances K^+^/Na^+^ homeostasis by reducing Na^+^ accumulation and preventing K^+^ depletion, thereby promoting salt tolerance [26]. Such imbalances also affect Ca^2+^ and Mg^2+^ uptake; for example, reduced Ca^2+^ levels weaken cell wall integrity, increasing susceptibility to necrosis in melon fruits and leaves [27]. In field-grown melons under saline irrigation, cultivars like ‘Sabouni’ show resilience through low leaf Na^+^ and high K^+^ concentrations, correlating with higher fruit yield and total soluble solids, whereas sensitive genotypes suffer from nutrient deficiencies that explain up to 92% of yield variation [27].

The interplay between ionic toxicity and nutrient imbalance in melons is organ-specific, with roots and leaves bearing the brunt of salt stress. In hydroponic experiments, salt-tolerant melon genotypes like CU 196 restrict Na^+^ translocation to shoots, storing toxic ions in older leaves to protect younger tissues, while maintaining higher K^+^ and Ca^2+^ concentrations in roots and stems [28]. This selective ion regulation prevents widespread nutrient deficiencies, such as K^+^ shortages that impair stomatal function and water use efficiency. Zhang et al. (2011) characterized a Shaker K^+^ channel (MIRK) in salt-tolerant melons that is inhibited by external Na^+^, reducing K^+^ influx in guard cells and potentially limiting Na^+^ arrival to shoots via stomatal closure [29]. Under drought-associated salinity, melons exhibit reduced hydric potential and altered amino acid profiles, with imbalances in isoleucine, glycine, and serine exacerbating ionic toxicity [3]. Cl^−^ toxicity, often overlooked, inhibits nitrate and phosphate absorption, leading to metabolic disorders like decreased dehydrogenase activity and further nutrient starvation [25]. In sensitive cultivars, leaf Na^+^/Ca^2+^ ratios increase, leading to cell membrane leakage and oxidative damage. Grafting onto tolerant rootstocks mitigates these effects by enhancing antioxidant defenses and regulating ion transport, thereby promoting K^+^ retention in shoots and Na^+^ efflux from roots [15]. Overall, these mechanisms underscore the genotypic variability in melons; tolerant varieties employ Na^+^ sequestration in vacuoles via NHX antiporters, preserving cytosolic K^+^/Na^+^ balance [25]. Field studies confirm that leaf ion content, particularly Na^+^ and K^+^, predicts melon yield under salinity, with heritability highest for traits like total soluble solids (TSS), which showed significant accumulation (a 30.7% increase) in salt-tolerant cultivars such as ‘Sabouni’ and ‘Shahabadi-1’, aiding breeding for resilience [27].

#### 2.3.3. Oxidative Damage

Salt stress induces oxidative damage in plants by disrupting ROS homeostasis, leading to the accumulation of superoxide (O_2_^−^), hydrogen peroxide (H_2_O_2_), hydroxyl radicals (·OH) and singlet oxygen (^1^O_2_) that impair cellular structures and functions [1,18,30]. In salt-sensitive genotypes such as CU 40 and CU 252, high salinity (200 mM NaCl) increases H_2_O_2_ levels, reducing stomatal conductance, leaf water potential, and biomass, while promoting pigment degradation and enzyme inactivation [20]. Similarly, Iranian landraces like “Kashan” exposed to 90 mM NaCl experience ROS over-accumulation, leading to declines in relative water content and membrane stability, along with nucleic acid damage, protein denaturation, and carbohydrate oxidation [31]. Salt stress also elevates malondialdehyde (MDA), an indicator of lipid peroxidation, causing membrane leakage and growth inhibition [32].

Mitigation of oxidative damage depends on genotypic variability and antioxidant defenses. Tolerant genotypes, including CU 159, CU 196, Suski-e-Sabz, Galia C8, and Midya cultivars, enhance activities of superoxide dismutase (SOD), catalase (CAT), peroxidases (POD) under salinity, reducing MDA accumulation, maintaining higher K^+^/Na^+^ ratios, and preserving growth, whereas sensitive varieties show minimal enzymatic response and severe oxidative injury [20,31]. Exogenous selenium supplementation could promote melon salt tolerance by boosting antioxidant activity, decreasing MDA and alleviating ROS damage, as seen in improved growth parameters [32]. Phytohormones also play a pivotal role in orchestrating these stress responses, with abscisic acid (ABA) emerging as a central regulator in melon abiotic stress adaptation, including salt stress [2,3,4]. ABA biosynthesis and signaling are rapidly upregulated in response to high salinity; for instance, the endogenous ABA level increases 8-fold in the Piel de Sapo melon cultivar exposed to salt stress [4]. The salinity-induced ABA formation not only triggers stomatal closure to minimize water loss but also plays a key role in salt stress tolerance by modulating osmoprotectant biosynthesis, ion homeostasis, and antioxidant defense, thereby enhancing cellular resilience [2,3,4].

## 3. Molecular Mechanisms of Salt Tolerance in Melon

Salt tolerance in plants is governed by a complex interplay of molecular processes that collectively maintain cellular homeostasis under saline conditions. These processes encompass ion transport and compartmentation, osmotic adjustment, reactive oxygen species (ROS) detoxification, hormonal regulation, and transcriptional control. In melon, emerging genomic and transcriptomic evidence indicates that both conserved stress-responsive pathways and species-specific regulatory mechanisms contribute to salinity adaptation. To elucidate these processes, it is essential to examine the molecular components that function at different hierarchical levels of stress response.

### 3.1. Ion Homeostasis Regulation

Maintaining low cytosolic Na^+^ levels and a high K^+^/Na^+^ ratio is crucial for plant salt tolerance. Plants employ two main strategies: active Na^+^ extrusion from the cytosol to the apoplast or soil via plasma membrane transporters and sequestration of Na^+^ into vacuoles by tonoplast antiporters. A conserved pathway, the salt overly sensitive (SOS) system, orchestrates this via the Ca^2+^-binding sensor SOS3 (a calcineurin B-like protein, CBL), kinase SOS2 (a CBL-interacting protein kinase, CIPK), and plasma membrane Na^+^/H^+^ exchanger SOS1, which activates Na^+^ efflux under salt stress [33,34]. Detailed mechanisms are well-characterized in model species like Arabidopsis and rice.

To date, several genes involved in ion transport regulation have been identified and functionally characterized in melon (Table 1). Among these, CmNHX1 was the first identified vacuolar Na^+^/H^+^ antiporter in melon, playing a key role in Na^+^ compartmentalization [35]. Its transcripts accumulate primarily in roots, stems, and leaves, with highest expression in roots under NaCl stress [35]. Heterologous expression of *CmNHX1* in the Na^+^-sensitive yeast mutant ATX3 enhanced salt tolerance, confirming its role in vacuolar Na^+^ sequestration and cytosolic detoxification. CmNHX1 functions analogously to AtNHX1 in Arabidopsis, contributing to vacuolar Na^+^ storage and osmotic balance [36]. Another critical regulator is CmHKT1;1, a plasma membrane transporter responsible for retrieving Na^+^ from the xylem and preventing excessive Na^+^ accumulation in shoots [37]. The expression of *CmHKT1;1* is markedly induced under salinity in tolerant melon cultivars, and overexpression of *CmHKT1;1* in Arabidopsis enhances salt tolerance by maintaining a favorable K^+^/Na^+^ ratio [37]. Functionally analogous to AtHKT1;1 and OsHKT1;5, CmHKT1;1 plays a conserved role in Na^+^ recirculation and shoot protection under salt stress [38,39].

Maintaining a high cellular K^+^/Na^+^ ratio is crucial for plant cells to tolerate salt stress, as it preserves ion homeostasis and protects essential metabolic processes. In melon, potassium channels are key regulators of this balance. CmSKOR, a Shaker-type outward-rectifying K^+^ channel, facilitates K^+^ efflux from root stelar cells to the xylem, thereby controlling K^+^ allocation to aerial organs [40]. Heterologous overexpression of *CmSKOR* in Arabidopsis has been shown to increase the cellular K^+^/Na^+^ ratio, enhancing plant tolerance to salt stress [40]. Melon Inward Rectifying K^+^ Channel (MIRK gene) is predominantly expressed in guard cells and vascular tissues, where it mediates K^+^ uptake [29]. By sustaining a favorable cellular K^+^/Na^+^ ratio under saline conditions, MIRK not only supports stomatal function but also contributes to overall salt tolerance in melon [29,41]. In addition to these channels, the HAK/KUP/KT (High-affinity K^+^ Transporters/K^+^ Uptake Permeases/K^+^ Transporters) family also plays a vital role in K^+^ transport and the maintenance of K^+^/Na^+^ homeostasis. In Arabidopsis, AtKUP/HAK/KT1, AtKUP/HAK/KT2, and AtKUP/HAK/KT6 have been reported to contribute to salt tolerance [42]. In melon, 14 *CmHAK* genes have been identified, yet their functional roles in conferring salt tolerance remain uncharacterized [43]. Comparative sequence analysis revealed that CmHAK12, CmHAK11, and CmHAK4 exhibit high sequence homology with Arabidopsis AtKUP/HAK/KT1, AtKUP/HAK/KT2, and AtKUP/HAK/KT6, suggesting that these melon HAK transporters may similarly contribute to the regulation of K^+^/Na^+^ homeostasis and salt stress adaptation [43].

**Figure 1:**
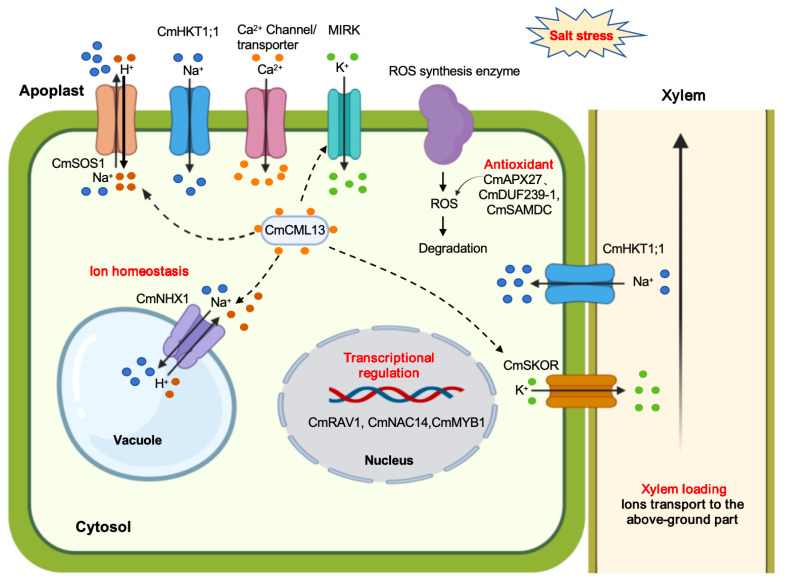
A model summarizing the molecular mechanism of salt tolerance regulation in melon. CmNHX1 is a vacuolar Na^+^/H^+^ antiporter that facilitates the sequestration of cytosolic Na^+^ into the vacuole. CmSOS1 maybe encodes a plasm membrane Na^+^/H^+^ antiporter that facilitates the sequestration of cytosolic Na^+^ out of cell. CmHKT1;1 serves as a sodium transporter responsible for retrieving Na^+^ from the xylem and preventing excessive Na^+^ accumulation in shoots. CmSKOR serves as a Shaker-type outward-rectifying K^+^ channel, facilitates K^+^ efflux from root stelar cells to the xylem. Melon Inward Rectifying K^+^ Channel (MIRK gene) mediates K^+^ uptake to keep favorable cellular K^+^/Na^+^ ratio under saline conditions. CmCML13 is a calmodulin-like (CML) protein and functions as a key Ca^2+^ sensor that maybe linking with CmNHX1, CmSOS1, CmSKOR and MIRK to regulate ion homeostasis. Melon ascorbate peroxidase CmAPX27, spermidine and spermine biosynthesis regulator CmSAMDC, and Domain of Unknown Function (DUF) family protein CmDUF239-1 alleviate salt stress-induced cellular damage by modulating antioxidant defense and reducing ROS accumulation. The transcription factor *CmRAV1*, *CmNAC14*, and *CmMYB1* also play an important role in melon under salt stress.

Calcium signaling serves as a pivotal regulatory mechanism that enables plants to perceive and respond to salt-induced ionic and osmotic disturbances. In melon, CmCML13, encoding a calmodulin-like (CML) protein, is markedly upregulated under salt stress [44]. Overexpression of *CmCML13* in Arabidopsis significantly enhances tolerance to both salt and drought stresses by limiting Na^+^ accumulation in shoots through an HKT1-independent pathway [44]. suggesting that CmCML13 functions as a key Ca^2+^ sensor linking stress perception with ion homeostasis regulation. Similarly, the expression of *CmCBL1*, *CmCIPK1-like* and *CmCIPK12-like*, is strongly induced by high NaCl concentrations in melon seedlings [45,46]. Altogether, these findings indicate that Ca^2+^-mediated signaling cascades, involving CMLs, CBLs, and CIPKs, cooperatively regulate downstream ion transport and maintain cellular homeostasis under salinity stress (Figure 1).

Multidrug and toxic compound extrusion (MATE) transporters exploit proton (H^+^) or sodium (Na^+^) gradients to mediate the efflux of diverse substrates, thereby contributing to ionic balance and pH homeostasis under salt stress [47,48]. In *Cucumis melo*, several *CmMATE* genes are transcriptionally induced by salt stress [49]. Although their specific functions have yet to be experimentally validated, phylogenetic and comparative analyses in other plant species suggest that these transporters may also facilitate ion and organic acid transport, thereby promoting detoxification, osmotic adjustment, and overall salt stress tolerance [47,48,49]. At the whole-plant level, these Ca^2+^ sensors and transporters act synergistically to regulate Na^+^ exclusion and K^+^ retention, enabling salt-tolerant melon genotypes to maintain higher K^+^/Na^+^ ratios compared with sensitive cultivars.

### 3.2. Antioxidant Regulation

Plants have developed an intricate antioxidant defense system composed of enzymatic and non-enzymatic components that act synergistically to detoxify ROS and preserve cellular redox balance [30]. The enzymatic machinery, primarily superoxide dismutase (SOD), catalase (CAT), peroxidases (POD), peroxiredoxins (PrxR) and ascorbate peroxidases (APX), catalyzes the stepwise conversion of ROS into less toxic molecules [30]. Meanwhile, non-enzymatic antioxidants, including ascorbate (ASC) and glutathione (GSH), function as redox buffers that sustain the cellular redox state and support the ASC-GSH cycle [30]. Physiological studies have demonstrated that the activities of SOD, CAT, and POD markedly increase in melon seedlings under salt stress, reflecting a rapid activation of enzymatic ROS scavenging pathways [5].

At the molecular level, several antioxidant-related genes have been functionally characterized in melon, shedding light on the genetic mechanisms underlying ROS regulation. CmAPX27, encoding an ascorbate peroxidase, enhances APX activity and strengthens ROS scavenging through the ASC-GSH pathway [50]. Its overexpression markedly improves salt tolerance and alleviates oxidative damage in transgenic plants [50]. Several *CmSOD* family members, including *CmMSD1, CmFSD2, CmFSD3, CmCSD1-1, CmCSD1-2*, and *CmCSD3*, are also transcriptionally induced by salt stress [51]. The upregulation of these genes enhances superoxide dismutation, thereby facilitating ROS detoxification and reinforcing the antioxidant defense system in melon. The *SAMDC* gene is a key regulator in the biosynthesis of spermidine and spermine, which play vital roles in free radical scavenging and oxidative stress mitigation [52,53]. Transgenic *Arabidopsis thaliana* plants overexpressing *CmSAMDC* significantly reduced MDA accumulation and markedly enhancing salt tolerance [54]. In addition, the Domain of Unknown Function (DUF) family proteins have been shown to mitigate ROS-induced damage by enhancing antioxidant enzyme activities and promoting the accumulation of osmolytes such as proline to sustain cellular water balance [55]. Specifically, CmDUF239-1 acts as a dual regulator of ion transport and ROS detoxification in melon [26,55]. Its overexpression increases the activities of antioxidant enzymes (SOD, POD, CAT), upregulates ion transport-related genes (*CmSOS1*, *CmNHX6*, *CmKUP3*, *CmSKOR*), and maintains K^+^/Na^+^ homeostasis under salt stress [26,55]. Collectively, these enzymatic- and non-enzymatic-mediated mechanisms constitute an integrated network that mitigates oxidative damage and supports melon adaptation to high salinity environments.

**Table 1:** Genes identified as regulators of salt stress in *Cucumis melo*.

Pathway	Gene	Function	Reference
Ion transport regulation	*MIRK*	MIRK mediates K^+^ uptake and maintains a favorable K^+^/Na^+^ balance under saline conditions.	[29,41]
	*CmNHX1*	CmNHX1, a vacuolar Na^+^/H^+^ antiporter, is mainly expressed in roots, stems, and leaves. Overexpression of *CmNHX1* in ATX3 yeast enhances salt tolerance.	[36]
	*CmHKT1;1*	CmHKT1;1 retrieves Na^+^ from the xylem to prevent excessive shoot accumulation and is strongly induced by salinity in tolerant melon cultivars. Overexpression in Arabidopsis enhances salt tolerance by maintaining a favorable K^+^/Na^+^ balance.	[37]
	*CmSKOR*	*CmSKOR* encodes a Shaker-type outward-rectifying K^+^ channel that mediates K^+^ efflux from root stelar cells to the xylem, thereby regulating K^+^ allocation to aerial tissues.	[40]
	*CmCML13*	*CmCML13* encodes a calmodulin-like (CML) protein, enhances salt tolerance in transgenic Arabidopsis by significantly reducing shoot Na^+^ content, independent of the HKT1 pathway.	[44]
Transcriptional regulation	*CmRAV1*	*CmRAV1* encodes a nuclear transcription factor with AP2 and B3 domains, is strongly induced by NaCl, especially in roots and flowers. Its overexpression in Arabidopsis enhances salt tolerance, improving germination and maintaining root growth under saline conditions.	[56]
	*CmNAC14*	*CmNAC14* acts as a negative regulator of salt stress, with its overexpression in Arabidopsis enhancing sensitivity.	[57]
	*CmMYB1*	*CmMYB1* respond rapidly to salt stress in early transcriptional reprogramming and negatively regulate salt stress.	[58]
Antioxidant regulation	*CmAPX27*	*CmAPX27* encodes an ascorbate peroxidase, enhances APX activity and strengthens ROS scavenging,	[50]
	*CmDUF239-1*	CmDUF239-1 plays a dual role in promoting salt tolerance by regulating antioxidant defenses and ion transport.	[26,55]
	*CmSAMDC*	CmSAMDC regulates spermidine and spermine biosynthesis, Overexpression of *CmSAMDC* in Arabidopsis enhances salt tolerance by reducing malondialdehyde (MDA) accumulation.	[54]
	*CmLEA-S*	CmLEA-S, a Late Embryogenesis Abundant (LEA) protein, protects cells by stabilizing membranes, scavenging ROS, and enhancing antioxidant enzyme activities.	[59]
Others regulation	*CmUBC*	Encodes an E2 ubiquitin-conjugating enzyme, constitutively expressed throughout diverse tissues and transcriptional induced by salinity.	[60]
	*CmKCS*	*CmKCS5*, *CmKCS6*, *CmKCS10*, and *CmKCS12* exhibit pronounced transcriptional upregulation under salt stress, likely bolstering melon membrane integrity.	[61]
	*CmTPR*	Several *CmTPR* genes are upregulated under salt stress, which is critical for maintaining protein stability and regulating stress-related pathways.	[62]

### 3.3. Transcriptional Regulation

Transcriptional regulation serves as a central hub in coordinating plant adaptive responses to salt stress, allowing rapid reprogramming of gene-expression networks that govern ion homeostasis, antioxidant defense, and osmotic adjustment. In melon *transcription factors* (*TFs*) from the *MYB, RAV*, and *NAC* families act as molecular switches that perceive salt-induced signals and modulate downstream genes involved in stress mitigation [4]. For instance, the *RAV*-family gene *CmRAV1* encodes a nuclear-localized transcription factor with AP2 and B3 DNA-binding domains [56]. Its expression is strongly induced by NaCl, especially in roots of salt-tolerant cultivars. Heterologous overexpression in Arabidopsis enhances seed germination, root elongation, and seedling survival under saline conditions [56]. Similarly, a genome-wide analysis identified 82 *CmNAC* genes encoding *NAC TFs* in melon, several of which are upregulated under salt stress, highlighting their role in salt tolerance. Among them, *CmNAC14* functions as a negative regulator, as its overexpression in *Arabidopsis* increases salt sensitivity [57]. In addition, members of the MYB TFs, such as *CmMYB1*, respond rapidly to salt stress in early transcriptional reprogramming; the overexpression of *CmMYB1* in *Arabidopsis* decreases plant salt tolerance, suggesting the gene negatively regulate salt stress [58]. These TFs operate within integrated regulatory networks that include Ca^2+^-sensor proteins, hormone signaling pathways, and epigenetic modifiers, allowing tissue- and development-specific control of stress responses [63]. For example, *CmRAV1* may coordinate with *CmDUF239-1* to co-regulate antioxidant enzyme genes, facilitating ROS detoxification while maintaining cellular redox balance [26]. In parallel, *TFs* influence ion transport and osmotic adjustment by regulating genes involved in K^+^/Na^+^ homeostasis, such as SOS1, NHX, and KUP family members, linking transcriptional control to physiological adaptation.

### 3.4. Other Molecular Pathways Associated with Salt Tolerance in Melon

Beyond ion homeostasis, antioxidant defense, and transcriptional regulation, melon employs additional molecular mechanisms to enhance salt tolerance, including lipid remodeling, hormonal and oxylipin signaling, and the activation of stress-protective proteins that collectively mitigate osmotic and oxidative damage. Ubiquitination is a major post-translational modification that selectively targets stress-damaged proteins for degradation via the 26S proteasome, thereby maintaining cellular proteostasis and preventing toxicity under salinity [60]. In melon, CmUBC, which encodes an E2 ubiquitin-conjugating enzyme, is ubiquitously expressed across all tissues and strongly induced by salt stress [60]. Its activity contributes to the clearance of damaged proteins, limits ROS accumulation, and reinforces antioxidant defenses, highlighting its central role in sustaining proteostasis under salinity conditions [64]. Membrane reinforcement via very-long-chain fatty acids (VLCFAs) vitally regulate plant salt tolerance, with β-ketoacyl CoA synthases (KCS) catalyzing VLCFA elongation to enhance cuticular wax and suberin deposition, thereby restricting Na^+^ influx [65]. Genome-wide analysis in melon identified 15 *CmKCS* genes distributed across eight chromosomes, among which *CmKCS5*, *CmKCS6*, *CmKCS10*, and *CmKCS12* are specifically upregulated under saline stress, likely contributing to the protection of the melon membrane system through modulation of VLCFA biosynthesis [61].

Several stress proteins also play crucial roles in maintaining cellular homeostasis under salinity stress. Late embryogenesis abundant (LEA) proteins, particularly the Y3SK2-type dehydrin CmLEA-S, protect cells by stabilizing membranes, scavenging ROS, and enhancing antioxidant enzyme activities, including APX and CAT [59]. Overexpression of *CmLEA-S* in transgenic tobacco significantly enhanced tolerance to high salinity [59]. Similarly, tetratricopeptide repeat (TPR) proteins function as molecular chaperones that facilitate protein folding, signal transduction, and stress-responsive protein–protein interactions [62]. Several *CmTPR* genes are upregulated under salt and drought conditions, implying their cooperative role in maintaining protein stability and regulating stress-related pathways [62].

## 4. Conclusions and Perspectives

*Cucumis melo* is an important economic crop widely cultivated worldwide for its unique flavor and high nutritional value. However, increasing soil salinity poses a serious threat to its sustainable production. High salinity disrupts melon physiology, inhibiting germination and seedling growth, reducing yield, and deteriorating fruit quality. Therefore, elucidating the mechanisms underlying salt tolerance and developing salt-tolerant melon cultivars are of great importance for sustaining and improving melon production.

Despite remarkable progress in uncovering the molecular basis of salt tolerance, considerable challenges remain before these findings can be effectively translated into breeding programs. Research on melon has investigated ion homeostasis, antioxidant defense, and transcriptional regulation, yet the systematic molecular mechanisms underlying salt tolerance remain poorly understood. Several genes (such as *CmNHX1*, *CmHKT1;1*, and *CmRAV1*, etc.) have been functionally characterized for their roles in Na^+^ compartmentalization or salt tolerance. However, the dynamic interactions of these genes with environmental factors, such as varying NaCl concentrations, ion compositions, and concurrent stresses like drought, remain largely unexplored, limiting predictive modeling for field performance. Moreover, the available melon germplasm for salt tolerance research remains critically limited, with most investigations relying on a narrow spectrum of cultivated varieties [4,66]. This restricted genetic base constrains the identification of novel alleles and hinders comprehensive quantitative trait locus (QTL) mapping for salt-responsive traits. The shortage of diverse, well-characterized accessions consequently narrows the genetic foundation necessary for developing robust salt-tolerant cultivars. Furthermore, the lack of integrative studies examining gene-environment interactions, including the modulation of salt tolerance mechanisms under fluctuating field conditions, restricts the effective translation of molecular discoveries into practical breeding applications [4,66]. Such limitations ultimately perpetuate productivity gaps in saline soils.

Looking ahead, future research on melon salt tolerance and breeding should adopt a more systematic and technologically integrated approach. Although numerous salt-responsive genes have been identified in melon, the comprehensive molecular framework underlying its salt adaptation remains largely unresolved. Integrating high-throughput omics approaches, such as transcriptomics, proteomics, and metabolomics, with CRISPR/Cas-mediated functional genomics will enable the precise identification of key regulatory genes [67,68], elucidate salt stress signaling and metabolic responses, and provide a solid foundation for breeding salt-tolerant melon cultivars. However, *Cucumis melo* suffers from very limited germplasm amenable to transformation and is highly genotype-dependent, making it difficult to find varieties that regenerate well [69,70]. Despite its relatively small genome (~450 Mb), the melon genome exhibits substantial structural variation and marked differences among subgenomic regions, contributing to strong genotype-dependent differences in regeneration and transformation competence [71,72]. In addition, melon is considered as a species that is recalcitrant to Agrobacterium-mediated transformation (AMT), with explant infection causing meristematic disruption, vacuolization, and low stable integration rates. Thus, genetic transformation in melon remains challenging [73]. Establishing a stable and efficient genetic transformation platform in melon is therefore crucial, as it would allow comprehensive functional characterization of salt-responsive genes and pathways and accelerate the translation of molecular insights into breeding programs for salt-resilient cultivars. Meanwhile, expanding germplasm resources by collecting wild relatives, landraces, and saline- or heat-adapted ecotypes is imperative to broaden the genetic base of melon. This diverse repository will facilitate genome-wide association studies (GWAS) and QTL mapping, enabling the identification of alleles that govern key salt-tolerance traits, including Na^+^ exclusion, K^+^ retention, and osmotic adjustment [31]. These genetic markers can be leveraged through targeted breeding strategies, such as marker-assisted selection (MAS) for precise introgression of specific QTLs and genomic selection (GS) for predicting breeding values across complex traits, to accelerate the development of high-yielding, salt-resilient varieties. Such insights are also essential for improving yield stability in arid and salinized environments. Finally, integrative studies should systematically link salt-tolerance genes with environmental variables, including NaCl gradients, ion compositions, and co-occurring stresses such as drought. Modeling gene-environment interactions enables researchers to assess the stability and adaptive plasticity of salt-tolerance traits, thereby integrating laboratory findings with field applications to breed cultivars resilient to diverse and challenging conditions. Together, these strategies will establish a robust framework for developing melon cultivars with enhanced salt tolerance and environmental resilience, contributing to sustainable production in saline soils.

## Data Availability

Data are contained within the article.
